# Spatial-temporal analysis of tuberculosis in Chongqing, China 2011-2018

**DOI:** 10.1186/s12879-020-05249-3

**Published:** 2020-07-22

**Authors:** Ya Yu, Bo Wu, Chengguo Wu, Qingya Wang, Daiyu Hu, Wei Chen

**Affiliations:** 1 Chongqing Institute of Tuberculosis Control and Prevention, Chongqing, China; 2 Chinese Field Epidemiology Training Program, Beijing, China; 3 National Center for Tuberculosis Control and Prevention, Chinese Center for Disease Control and Prevention, Beijing, China

**Keywords:** Epidemiology, Spatial analysis, Tuberculosis

## Abstract

**Background:**

China is a country with a high burden of pulmonary tuberculosis (PTB). Chongqing is in the southwest of China, where the notification rate of PTB ranks tenth in China. This study analyzed the temporal and spatial distribution characteristics of PTB in Chongqing in order to improve TB control measures.

**Methods:**

A spatial-temporal analysis has been performed based on the data of PTB from 2011 to 2018, which was extracted from the National Surveillance System. The effect of TB control was measured by variation trend of pathogenic positive PTB notification rate and total TB notification rate. Time series, spatial autonomic correlation and spatial-temporal scanning methods were used to identify the temporal trends and spatial patterns at county level.

**Results:**

A total of 188,528 cases were included in this study. A downward trend was observed in PTB between 2011 and 2018 in Chongqing. The peak of PTB notification occurred in late winter and early spring annually. By calculating the value of Global Moran’s *I* and Local Getis’s *G*_i_^*^, we found that PTB was spatially clustered and some significant hot spots were detected in the southeast and northeast of Chongqing. One most likely cluster and three secondary clusters were identified by Kulldorff’s scan spatial-temporal Statistic.

**Conclusions:**

This study identified seasonal patterns and spatial-temporal clusters of PTB cases in Chongqing. Priorities should be given to southeast and northeast of Chongqing for better TB control.

## Background

Tuberculosis (TB) is a chronic infectious disease caused by *Mycobacterium tuberculosis* (MTB). It is one of the top 10 causes of death [1]. China contributed 9% of total cases in the world in 2017, ranking the second place after India [1]. In the 1990s, a TB control project, including directly observed treatment and short-course (DOTS) strategy, was implemented in 13 provinces of China [2]. Then the strategy had been expanded to the whole country by 2005 [3]. After more than 10 y efforts, China has reached the 2005 global tuberculosis control targets [4]. It is the only country with a high TB burden that has made the achievements [5]. But TB remains a major public health problem in China, especially in the relatively poor northern and western areas [6, 7].

Chongqing is located in the southwest of China with 39 districts/counties, and 14 of them are national-level poor districts and counties [8]. In 2018, about 20,000 TB cases were reported in Chongqing with the overall notification rate of 73.4 cases per 100,000 population, which was higher than the average of the whole country [9]. A previous study of Chongqing pointed out that distribution of TB was uneven across the city [10], and a certain degree of seasonality was observed [11].

In recent years, spatial-temporal analysis has been widely used to describe the distribution characteristics and transmission patterns of tuberculosis in China [7, 12, 13] and other countries [14–18]. These studies demonstrated that TB has a highly complex dynamics and is spatially heterogeneous at provincial, national, and international levels during certain periods of time [19]. Few studies have been conducted in Chongqing to explore the spatial epidemiology at the county level. In order to improve TB control measures, we conducted Geographical Information System (GIS) based spatial-temporal scan statistic in Chongqing from 2011 to 2018.

## Methods

### Study setting

Chongqing is a mountainous city located in southwest China, with 27 districts and 12 counties. It covers an area of 82,400 km^2^ [20], of which 76% are mountains, 22% hills, 2% valleys and plains. The main stream of the Yangtze River runs through the city from west to east, with a flow of 665 km. In 2018, there are 31 million permanent residents in Chongqing, of whom about 20 million were urban population, accounting for 65.5% of total residents. The gross domestic product (GDP) per capita was RMB 65,933 Yuan in 2018 [21], which was 1.9 times higher than that of 2011.

### Data collection and management

The Chongqing TB surveillance data from January 2011 to December 2018 was extracted from the National Surveillance System for Notifiable Infectious Disease, which is established and operated by the Chinese Center for Disease Control and Prevention (CCDC). This surveillance system covers all counties of 32 provinces, autonomous regions and municipalities of China, and collects data of all TB cases reported by hospitals. We obtained these TB data from Chongqing Institute of Tuberculosis Control and Prevention, and were permitted to use by CCDC.

Each case contains demographic information as well as medical information. Each case is identified by the unique identity card (ID) number to avoid duplicate reports. In order to protect patients’ privacy, asterisk was used to block the name and ID number of all cases when the data were extracted.

The effect of TB control was measured by the variation trend of pathogenic positive PTB notification rate and total TB notification rate. Pathogenic positive PTB includes smear positive (SS+) cases and smear negative but culture positive (S-C+) cases. With the improvement of national infectious disease surveillance system, the rifampicin resistant tuberculosis (RR-TB) information has been added to the system since July 1, 2017 [22], so pathogenic positive PTB in 2017 and 2018 also included RR-TB cases. The overall PTB included: 1) pathogenic positive PTB; 2) pathogenic negative PTB, referring to those PTB patients whose smear and culture were both negative; and 3) PTB without pathogenic examination, referring to those PTB patients who had slight symptom and could not cough up with sputum.

Each enrolled case was geocoded by current address. Then them were matched to the county-level polygon maps of the geographic information (Geographic database from China CDC) at a 1:1,000,000 scale as the layer’s attribute table by the same identified number. The longitude and latitude coordinates of the central point for each district and county were located though the Google geocoding service and the toolbox of Geoprocessing in ArcGIS v.10 (ESRI Inc., Redlands, CA, USA). The coordinate information was used for spatial-temporal analysis. This geocoding process has been widely applied in previous studies [7, 19, 23, 24], and we used a similar approach.

The annual population data of each administrative district from 2011 to 2018 were obtained from the Chongqing Statistical Yearbook and the Basic Information System for Disease Prevention and Control.

### Statistics analysis

#### Time series and descriptive analysis

The epidemiological characteristics of TB cases were analyzed at provincial level. TB cases reported from 2011 to 2018 were aggregated by age, gender, occupation and date of onset for TB notification rate analysis. Comparison between different demographic groups was carried out by Chi-square test or Fisher’s exact test. The trend of the notification rate was tested using Cochran-Armitage test. All statistical analyses were performed using SPSS 22.0 (SPSS Inc., Chicago, IL, USA). The difference was considered significant if *P*-value was less than 0.05.

The temporal patterns were examined by looking at the onset month of all TB cases. The time series included 96 months from January 2011 to December 2018 and was examined using EXCEL2016.

#### Spatial autocorrelation analysis

Spatial autocorrelation analysis is a spatial statistical method that can reveal the regional structure of spatial variables. It can verify whether an element attribute value is associated with an attribute value at an adjacent space point. It mainly includes global autocorrelation analysis and local autocorrelation analysis.

Global Moran’s *I* values [25] calculated by ArcGIS v.10 software (ESRI Inc., Redlands, CA, USA) was used to identify spatial autocorrelation and detect the spatial distribution pattern of TB in Chongqing, China. The range of Moran *I* value is between − 1 and 1. A positive Moran *I* value indicates that a positive correlation exists, and the larger the value, the more obvious the tendency to cluster is, while a negative Moran *I* value indicates that a negative correlation exists, showing a discrete distribution. There is no spatial clustering when the value is zero, meaning that the data are randomly distributed [26]. Both *Z*-score and *P*-value are used to evaluate the significance of Moran’s *I* [27].

Local Getis’s *G*_i_^*^ statistic [28] is used to identify the local level of spatial autocorrelation and determine locations of clusters or hotspots. Value of *G*_i_^*^ in this study was calculated using ArcGIS v.10 software (ESRI Inc., Redlands, CA, USA).

#### Spatial-temporal scan statistic

Kulldorff’s spatial-temporal scan statistical analysis was used to identify the spatial, temporal and clusters of PTB across different counties geographically and in different time period. SaTScanTM version 9.1.1 software (Kulldorff, Boston, MA, USA) was used based on the Poisson probability model [29]. The SaTScan™ software was developed by Martin Kulldorff together with Information Management Services Inc. and could download on Web site (http://www.satscan.org/).

This method is based on creating a moving cylinder that contains geographical information with height corresponding to time [30]. The maximum radius of the bottom of the cylinder, which is the maximum area of scanning, was set in this analysis at 50% of the total population and the height corresponding to the time of the study area. Log likelihood ratio (LLR) of different circle centers and different radii was calculated to compare the notification rate of TB within the circular window and outside the circular window [31]. The larger the LLR value was, the more it likely to be the cluster. Monte Carlo simulation test was used to evaluate whether the difference is statistically significant. For each possible spatial-temporal cluster, when the *P*-value is less than 0.05, a higher LLR value indicates that the area covered by this dynamic scanning window is more likely to be a cluster region. The window with the largest LLR value is most likely a cluster and the secondary clusters are the other windows with statistically significant LLR value. Finally, ArcMap software was used to visualize the scanning results.

### Ethical review

The TB data of this study were used with the approval of Ethics Committee of the Institute of Tuberculosis Control and Prevention in Chongqing. The personal identifiable information of each case in our data analysis has been deleted, and the availability of the data set is still restricted. However, with the permission of the National Center for Tuberculosis Control and Prevention (NCTB) under CCDC, the data is accessible from the corresponding author.

## Results

### Descriptive analysis of PTB cases

**Fig. 1:**
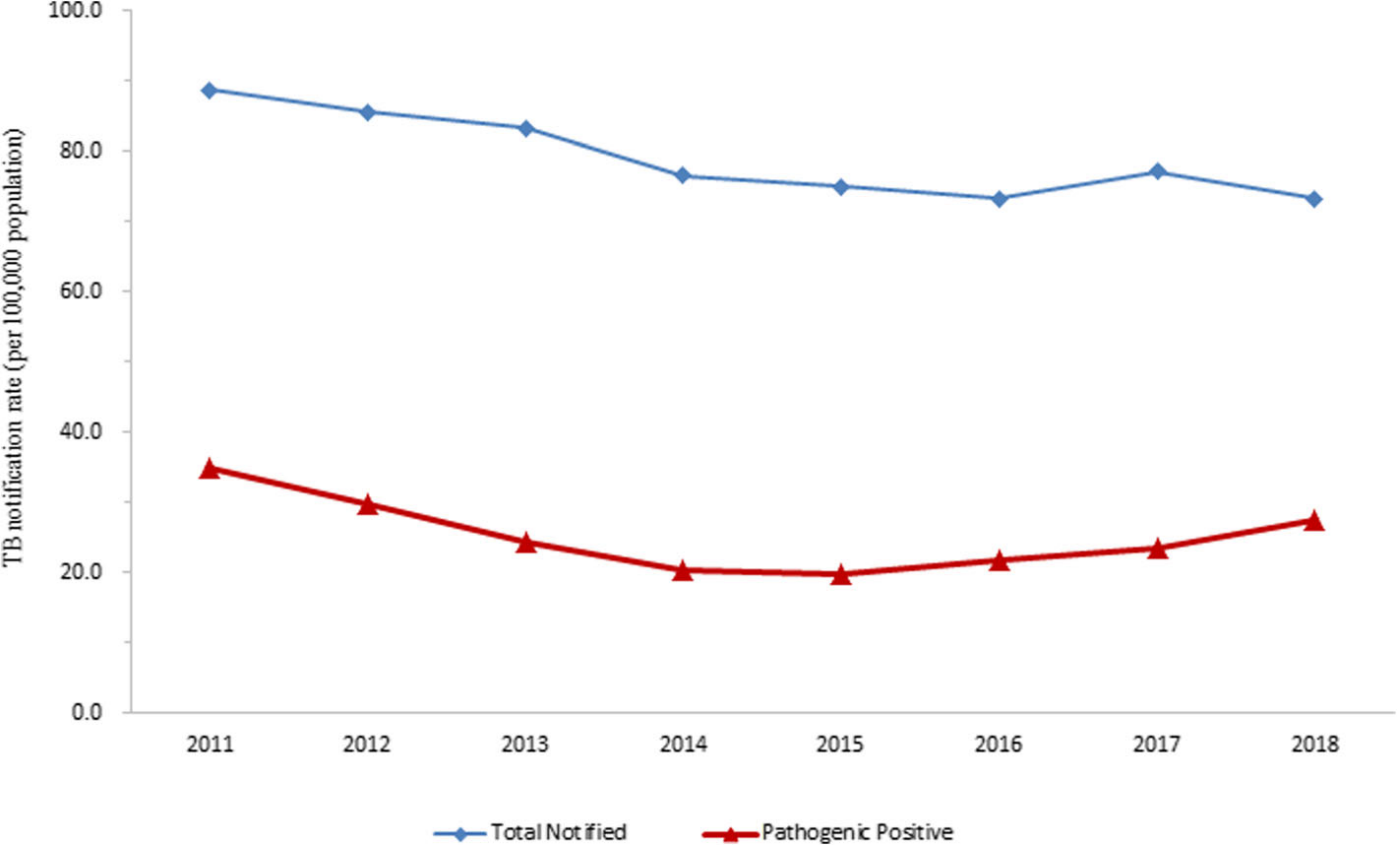
The variation trend of TB notification rate from 2011 to 2018

A total of 188,528 PTB cases were notified in Chongqing from 2011 to 2018. Among them, 32% (60,254 cases) were pathogenic positive. From 2017 to 2018, 910 cases of RR-TB were reported. The annual average notification rate of all PTB and pathogenic positive PTB were 79 cases per 100,000 population and 25 cases per 100,000 population, respectively. The total PTB notification rate decreased significantly from 88.8 cases per 100,000 population in 2011 to 73.4 cases per 100,000 population in 2018 (*χ*^2^ trend = 732.178, *P* <  0.001) (Fig. 1). The annual average notification rate of male was significantly higher than that of female (*χ*^2^ = 30,273.043, *P* <  0.001). The notification rate of PTB was the highest among people over 60 years of age. More than half of the PTB patients were farmers, and the average proportion of farmer PTB patients was 54.3% between 2011 and 2018. The demographic characteristics of the PTB cases in Chongqing from 2011 to 2018 were shown in Table 1.

**Table 1:** Notification rates of PTB with different demographic characteristics in Chongqing from 2011 to 2018, (1/100,000)

Characteristics	2011	2012	2013	2014	2015	2016	2017	2018
Gender								
Male	124.8	120.2	116.6	106.5	103.7	102.8	107.5	102.6
Female	51.9	50.2	49.2	46.0	45.6	43.1	46.0	43.6
Age (year)								
0–14	7.2	5.8	5.5	4.4	4.6	4.0	4.3	4.5
15–29	119.9	108.0	105.2	96.8	90.7	88.6	96.2	87.3
30–44	80.5	85.5	80.2	70.5	65.4	60.7	59.8	54.1
45–59	109.4	96.9	88.1	82.0	82.0	82.5	84.2	83.7
≥ 60	124.2	119.7	125.5	117.4	121.3	115.6	132.8	127.9
Occupation								
Student	38.1	34.2	34.5	33.2	29.6	31.0	36.1	37.5
Teacher	66.4	50.2	54.2	47.8	39.3	42.7	40.2	45.1
Medical staff	90.0	116.5	85.8	110.4	85.7	97.7	93.1	85.2
Farmer	115.6	113.6	113.3	107.2	110.0	109.3	109.4	100.7
Others	79.2	76.7	72.9	64.1	60.7	58.1	66.8	66.2

### Temporal patterns of PTB cases

**Fig. 2:**
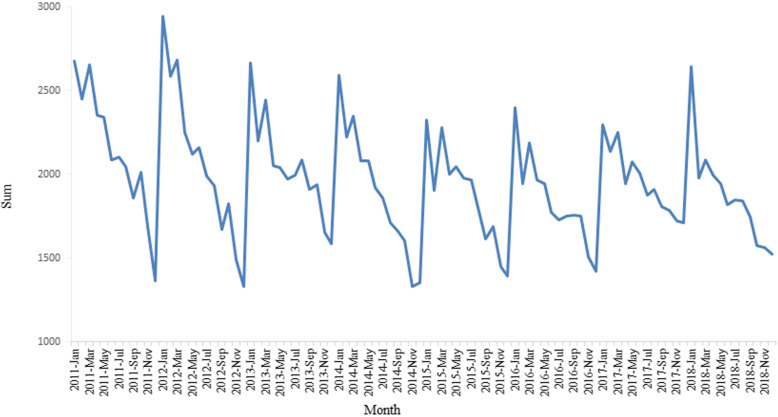
The monthly fluctuation of PTB cases in Chongqing from 2011 to 2018

An obviously temporal trend variation of PTB cases in Chongqing from 2011 to 2018 was showed in Fig. 2. The number of PTB peaked in the January and March each year, then showed a volatile downward trend after March, and declined to nadir in December.

### Spatial patterns of PTB cases

**Fig. 3:**
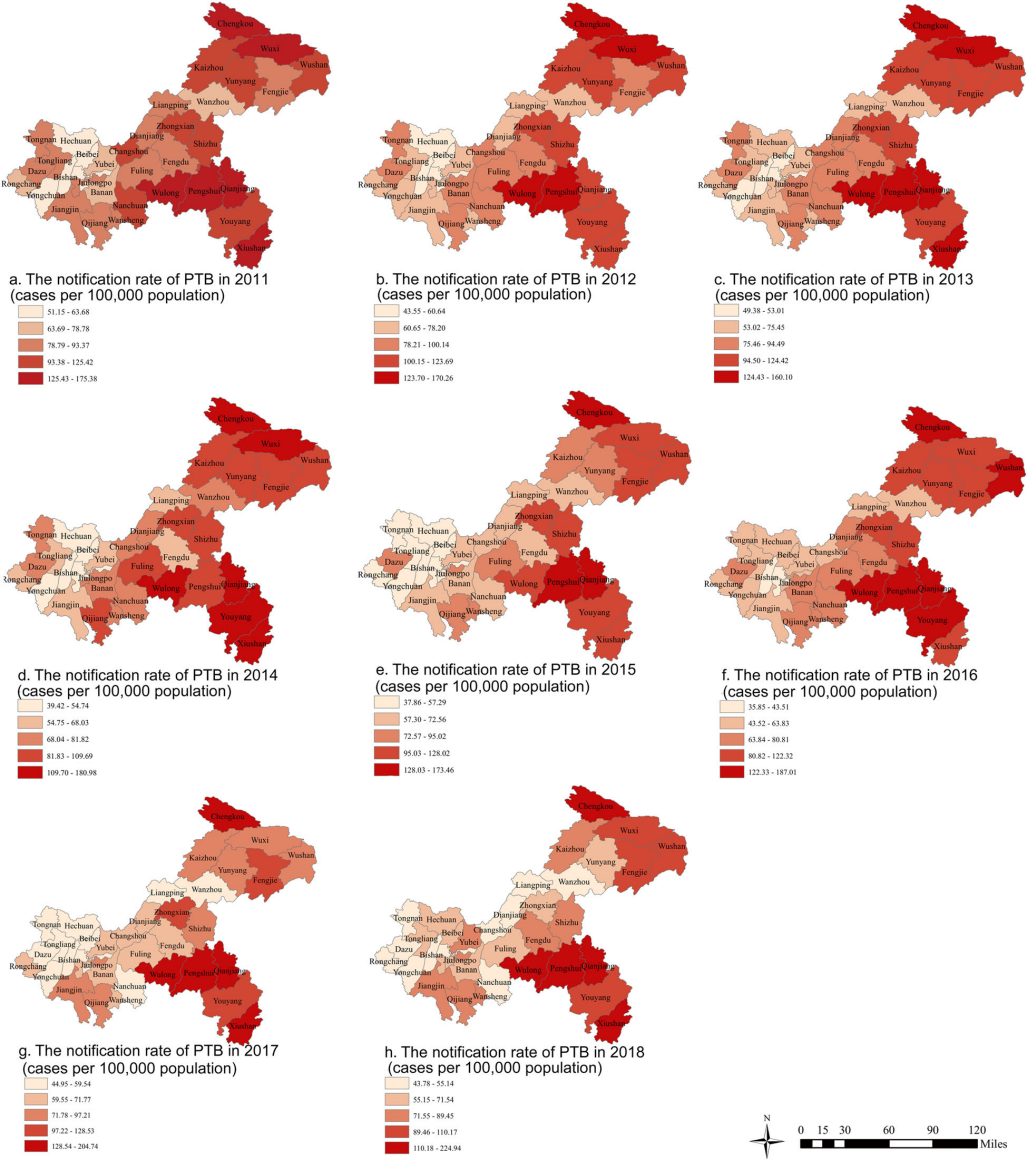
The notification rates of PTB at county level in Chongqing, 2011–2018. The map depicted in the figure is our own

The spatial variations of PTB notification rates between 2011 and 2018 at county level in Chongqing were showed in Fig. 3. The highest notification rates were found in Pengshui county, Xiushan county, Chengkou county, Qianjiang district and Wulong district.

The global spatial autocorrelation analysis showed that the annual Moran’s *I* values of PTB notification rates from 2011 to 2018 were significantly different (Table 2), indicating that the notification rates of PTB in Chongqing were non-randomly distribution, and the distribution of PTB was spatially autocorrelated in Chongqing over the period of 8 y. The Moran’s *I* values of annual PTB notification rates were positive, and the *P* values were less than 0.001. It pointed out that there was a positive global spatial autocorrelation in the notification rates of PTB each year.

**Table 2:** Global spatial autocorrelation analyses for annul PTB notification rate in Chongqing, China from 2011 to 2018

Year	Moran’s *I*	*Z*-score	*P* value
2011	0.489155	5.182869	< 0.001
2012	0.485119	5.139183	< 0.001
2013	0.594580	6.165923	< 0.001
2014	0.519618	5.541707	< 0.001
2015	0.577399	6.083351	< 0.001
2016	0.551651	5.847773	< 0.001
2017	0.413744	4.493974	< 0.001
2018	0.365869	4.079302	< 0.001

**Fig. 4:**
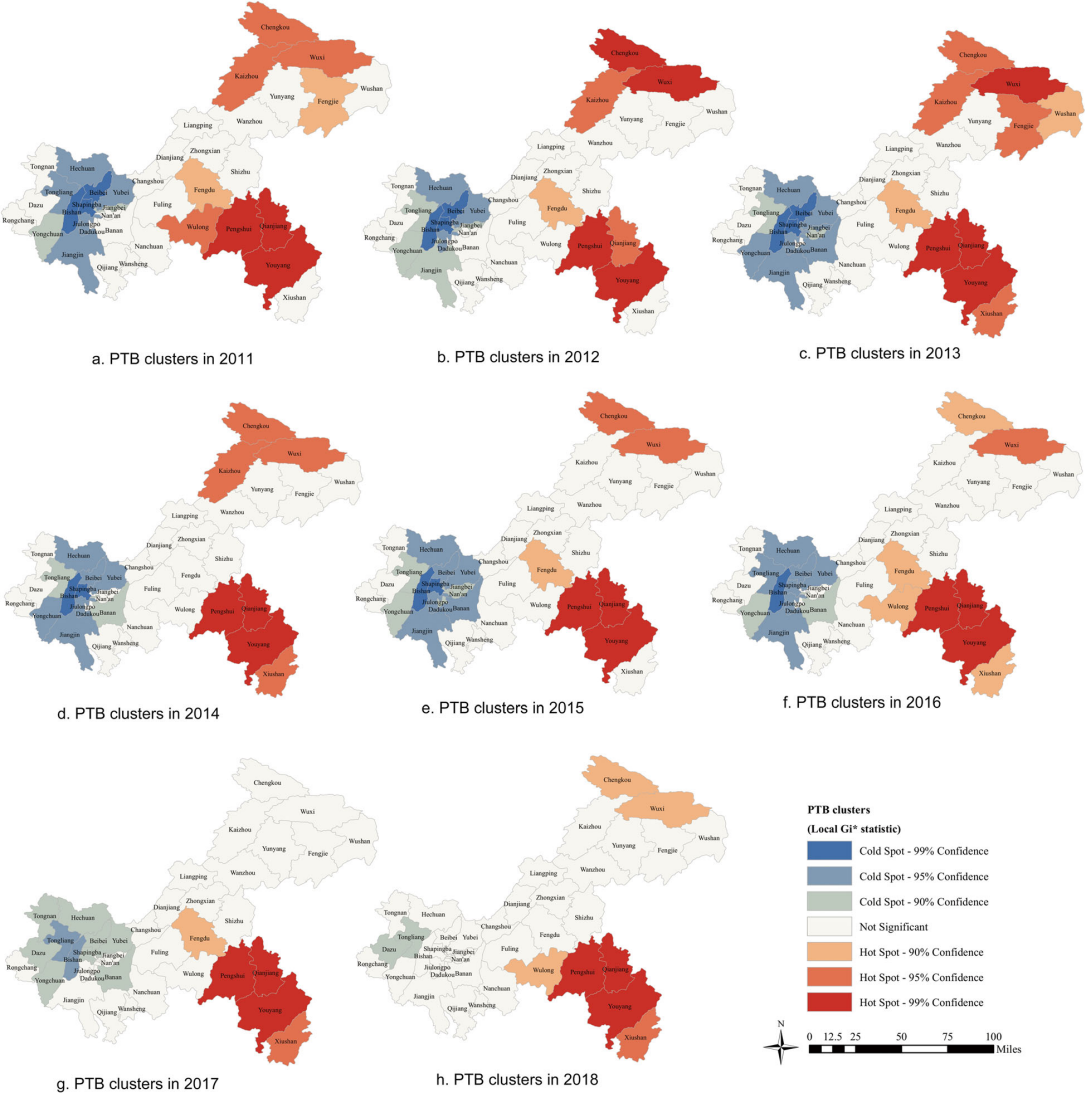
The spatial clusters of the PTB cases at the county level using the Local *G*_i_^*^ statistic. The map depicted in the figure is our own

Figure 4 showed the analysis results of the local spatial autocorrelation. The clusters of PTB cases, including hot spots and cold spots, were identified by using Local Getis’s Gi* statistic. On the whole, the hot spots of PTB in Chongqing from 2011 to 2018 were concentrated in the southeast and northeast regions. It indicates that the notification rate of PTB was high in some districts and counties and their surrounding areas. All these districts and counties with high notification rate covered most areas of southeastern and northeastern regions of Chongqing. But a small number of hot spots had changed dynamically. From 2011 to 2018, three areas in the southeast part of Chongqing, such as Pengshui county, Youyang county and Qianjiang district, had always been the hot spots of PTB. However, there were slight changes in the hot spots of PTB in northeastern Chongqing each year. As time went by, the hotspots in the northeast gradually narrowed till they disappeared in 2017. But two hotspots appeared again in the northeast in 2018. On the other hand, the main urban zone and the western region were the cold spots of TB. Bishan, Shapingba and Beibei districts were the three most significant cold spots of PTB in the city. But in 2018, the aggregation of the cold spots was not so obvious.

### Spatial-temporal clustering analysis by SaTScan

**Fig. 5:**
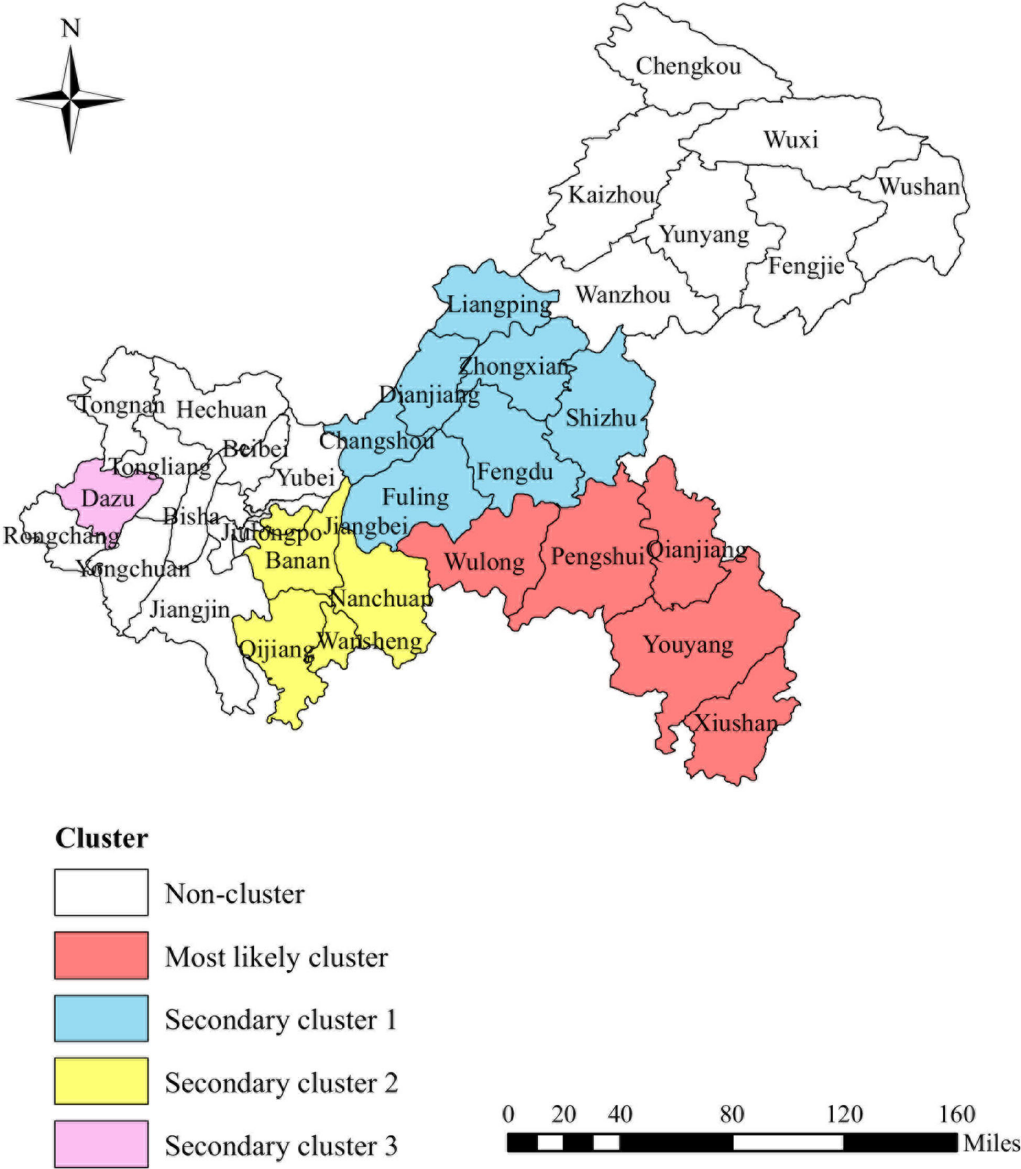
The space-time clusters of PTB cases at the county level in Chongqing, 2011–2018. The map depicted in the figure is our own

The notification rates of PTB during 2011–2018 were analyzed with spatial-temporal scanning. The results showed that the notification rates of PTB were spatial-temporal clustered. One most likely cluster and three secondary clusters were been showed in Table 3 and Fig. 5.

**Table 3:** Significant high-rate PTB Clusters in Chongqing, China detected by SaTScan from 2011 to 2018

Cluster Type	Number of Clustering areas	Cluster districts and counties	Time frame	Observed cases	Expected cases	Relative risk	Log likelihood ratio	*P* value
Most likely cluster	5	Xiushan, Youyang	2015–2018	14,787	7427.77	2.08	2974.12	< 0.01
		Qianjiang, Pengshui						
		Wulong						
Secondary cluster 1	7	Dianjiang, Changshou	2011–2013	13,885	11,925.43	1.18	163.79	< 0.01
		Liangping, Fengdu						
		Fuling, Zhongxian						
		Shizhu						
Secondary cluster 2	5	Qijiang, Wansheng	2011–2012	5693	5212.56	1.1	22.12	< 0.01
		Banan, Nanchuan						
		Nan’an						
Secondary cluster 3	1	Dazu	2012–2013	1337	1153.37	1.16	13.99	< 0.01

The most likely cluster was mainly distributed in the southeast of Chongqing, which covered three counties and two districts: Xiushan county, Youyang county, Pengshui county, Qianjiang district, and Wulong district. The clustering time was from January 2015 to December 2018. A total of 14,787 cases were identified during this period. Our analysis results showed that the risk of PTB in these districts and counties was 2.08 times higher than that outside the hot spots. In addition, three statistically significant secondary clusters were also detected with high incidence of TB. These three secondary clusters distributed in different areas of Chongqing. They were seven districts and counties in the central area, five districts and counties in the southwest area and one district in the western area. And the clustering time were from January 2011 to December 2013, January 2011 to December 2012, and January 2012 to December 2013, respectively.

## Discussion

In our study, we first made a descriptive analysis of the epidemic situation of PTB in Chongqing; then we used the time series method to analyze the temporal patterns of PTB cases; finally, we used the spatial analysis method to study the spatial patterns and spatial-temporal clustering at the county level. The notification rate of PTB in Chongqing decreased steadily during the eight-year study period. The PTB notification rate had declined to 73.4 cases per 100,000 population in 2018 from the peak of 88.8 cases per 100,000 population in 2011. This downward trend was consistent with the studies of other provinces and cities, and of the whole country [7, 12, 23, 32]. It shows that the prevalence of PTB in Chongqing has been controlled to a certain extent. This achievement is due in large part to the Chongqing Municipal Government and the Health Administrative Department that have attached great importance to TB control and prevention in recent years, and have increased investment needed. First, the government has issued some good policies. For instance, free treatment of TB patients has been included in the pro-people projects [33] in which the municipal government would be assessed periodically. Following that, the Tuberculosis Control and Prevention Plan (2011–2015) was issued in February 2012 and effective measures have been implemented. Second, TB control funding has increased continuously. In 2018, the investment for TB control and prevention in Chongqing was RMB 1.03 per capita, which was approximately twice as much as the national average, making Chongqing rank the sixth among all the provinces in China. Third, at the request of the Chongqing Municipal Tuberculosis Control Institute, Chongqing Municipal Health Commission has purchased a batch of molecular biology testing equipment and reagents for TB diagnosis and treatment, and distributed them to designated TB hospitals in all districts and counties of the city. As a result, the diagnostic time of PTB has shortened and the possibility of pre-treatment transmission has decreased. Meanwhile, rifampicin resistance testing was performed on TB patients. After the implementation of these effective measures, the TB epidemic situation in Chongqing has improved significantly, though Chongqing is still one of the cities with high TB burden in China [34].

Through time series analysis, we found that the TB epidemic in Chongqing showed an obvious cyclical trend. TB onset was mainly concentrated in the first half of each year, especially between January and March. Chongqing has a subtropical monsoon humid climate. The average temperature in the coldest months is four to eight degrees Celsius, and the average annual relative humidity is 70 to 80%. It is one of the regions with the least annual sunshine in China. To the best of our knowledge, lack of exposures to ultra violet from sunlight and the poor ventilation in indoor settings may increase the opportunity of infecting TB bacteria [35]. In late winter and early spring in Chongqing, residents close doors and windows, and reduce outdoor activities because of the cold, foggy and damp weather. There may be two other reasons for this temporal trend. One was that many residents received free TB screening in the spring because of massive public information campaigns aiming at controlling TB before and after the World TB Day. Another reason was that March was the back-to-school month, and students were tested for TB according to government requirements. The reason for the decrease in patients reported in February is that the Chinese Spring Festival usually falls in February, in which people are busy celebrating the lunar New Year, and avoiding to seek medical care. This seasonal pattern is the same as that of a national study [12, 13], and studies in Guangxi province [36], Xinjiang Uygur Autonomous Region [37], Zhaotong city of Yunnan province [24, 32] and other western regions, but a little different from that of studies in Zhejiang province [23], Hong Kong [38] and Taiwan [39], which are located in the eastern coastal area.

The field of spatial epidemiology has evolved rapidly in the past two decades [40, 41], and it has been widely used in the study of TB [42, 43]. The global spatial autocorrelation results in this study indicated that PTB in Chongqing shows an obvious spatial clustering distribution. And advanced local spatial autocorrelation analysis showed that the hot spots of PTB had a slight dynamic variation over time. The TB hotspots detected in this study are basically consistent with the notification rates of PTB in Chongqing. Pengshui county, Qianjiang district, Wulong district, Xiushan county and Youyang county located in the southeast of Chongqing, were the areas with high notification rates of PTB in the past 8 y. Their annual average notification rates from 2011 to 2018 ranks first, second, fourth, fifth and seventh, respectively. Chengkou and Wuxi counties located in the northeast of Chongqing were also hotspots, except in 2017. The following facts may explain why these counties and districts became hot spots. First of all, these counties and districts were poor areas with backward economy and shortage of health resources. Their GDP was the lowest in Chongqing. Most of the residents living there are ethnic minorities. Lack of funds and professionals for TB control has constrained the implementation of local TB control program. In addition, people living in these poor counties often have no fixed income and cannot afford the diagnosis and treatment of TB, leading to the continued spread of the disease. All these were the reasons of PTB clustering in adjacent areas [44]. Some changes, however, merit attention. For example, Kaizhou district has been removed from the hot spots since 2015. This indicates that the notification rate of PTB in Kaizhou district was significantly lower than that of neighboring hotspots. The reason for the decline of TB epidemic in Kaizhou district was that the local government has paid more and more attention to TB control. The local government had implemented a series of interventions that were not taken by other districts. First of all, the government sent officials to conduct field investigations on local TB control work, and issued such policy documents as 5-year planning, standardized implementation, and performance appraisal of TB control program. In these documents it was required that every PTB patient must be identified and cured. Besides, extensive health education and TB screening for key groups should be strengthened. The key groups were close contacts of TB patients, people living with HIV (PLHIV), the elderly, students and floating population. The government committed to reward units and individuals that have made significant contributions to local TB control. Second, a total of RMB 8 million has been invested in local TB control since 2014, including over RMB 5 million special funds, RMB 500,000 for salvaging poor patients, RMB 450,000 for TB screening for the second grade students of senior high schools, and more than RMB 2 million for public health institutions and personnel. Third, the introduction of talents has been enhanced. For instance, more than 10 new professionals for TB control were recruited in 2015. Fourth, new equipment for rapid diagnosis has been purchased, and an electronic medical record system for patient medication management has been developed. Finally, education department and mass media have been involved to strengthen the promotion of TB control. After the implementing all these measures, Kaizhou district has achieved remarkable improvement in TB control. Based on the results of global and local spatial analysis, we have initially identified that the southeastern region and the northeast region are the important regions for TB control and prevention in Chongqing.

Taking the role of time factors in the geographical distribution of diseases into consideration, we used spatial-temporal scanning analysis to supplement the simple spatial analysis. In the previous analysis of the spatial-temporal clustering characteristics of the national TB epidemic, we found that Chongqing was in the most likely cluster [7] from 2005 to 2011, and in another study, Chongqing was in the secondary cluster from 2005 to 2015 [13]. These results show that although the burden of PTB in Chongqing is declining gradually, it is still high. A spatial-temporal scan analysis of the PTB cases from 2011 to 2018 showed that the most likely cluster was concentrated in the southeast of Chongqing, covering two districts and three counties. The clustering time period was from 2015 to 2018. Obviously, it indicated that these counties and districts bore excess burden of PTB and had higher risk of disease transmission, so they are the most important areas for TB control in the next few years. More effective, stronger and targeted measures should be implemented to control TB transmission in these areas. During the study period, no spatial-temporal cluster was detected in the northeast region, which also showed that the TB prevention and control work in these regions has achieved satisfactory results since the implementation of the Eleventh Five-year Plan. However, according to the local autocorrelation analysis, there were still scattered hotspots in the northeastern region. Hence the TB control in these areas should not be relaxed.

This is the first time to analyze the spatial-temporal clustering characteristics at county level in Chongqing. The results of the analysis helped us to identify the high risk areas of PTB in the city. This is very important for our next step in TB control.

There are some limitations in the study. First, our analysis was based on data obtained from the National Surveillance System, so it is possible that a small number of cases had not been captured, which might cause underestimation of PTB epidemic in Chongqing. Second, the geographical information of townships (the smallest unit of administrative division) of each district and county was not available, so we did not analyze the temporal and spatial characteristics of PTB at township level. Third, the notification of RR-TB may not be adequate because not all TB patients were tested for rifampicin resistance, so we did not analyze the characteristics of RR-TB. Finally, potential risk factors, such as poverty [45], low education level [46], poor living conditions [47, 48], inadequate access to medical services [49] and environmental pollution [50], which previously has been reported to be associated with a high incidence of TB, were not evaluated in this study. We will consider narrowing the geographical scale to the township level and incorporating relevant risk factors for further analysis.

## Conclusions

This study identified seasonal patterns and spatial-temporal clusters of PTB cases at the county level in Chongqing from 2011 to 2018. The most likely clustering time was spring, and the most likely clustering areas were southeast and northeast regions of Chongqing. The spatial-temporal clustering results by SaTScan showed that southeastern Chongqing had higher TB burden and risks of TB transmission after 2015. Priorities should be focused to these areas in subsequent TB control measures.

## Data Availability

The data of TB patients that support the findings of this study cannot be shared publicly because the data contain sensitive patient information, and sharing local sensitive contagious disease data publicly without license is illegal. The Ethics Committee of the Institute of Tuberculosis Control and Prevention in Chongqing has imposed this restriction. Data are available from corresponding author for researchers who meet the criteria for access to confidential data.

## Abbreviations

PTB: Pulmonary tuberculosis
TB: Tuberculosis
MTB: *Mycobacterium tuberculosis*
DOTS: Directly observed treatment and short-course
GIS: Geographical Information System
GDP: Gross Domestic Product
CDC: Center for Disease Control and Prevention
ID: Identity card
SS+: Smear positive
S-C+: Smear negative but culture positive
RR-TB: Rifampicin resistant tuberculosis
LLR: Log likelihood ratio
NCTB: National Center for Tuberculosis Control and Prevention
HIV: Human immunodeficiency virus

## References

[1] World Health Organization. Global Tuberculosis Report. Geneva: the World Health Organization. 2018. https://apps.who.int/iris/bitstream/handle/10665/274453/9789241565646-eng.pdf?sequence=1&isAllowed=y. Accessed 14 Mar 2020.

[2] China Tuberculosis Control Collaboration (1996). Results of directly observed short-course chemotherapy in 112,842 Chinese patients with smear-positive tuberculosis. China Tuberculosis Control Collaboration. Lancet.

[3] Wang L, Zhang H, Ruan Y, Chin DP, Xia Y, Cheng S (2014). Tuberculosis prevalence in China, 1990-2010; a longitudinal analysis of national survey data. Lancet.

[4] Dye C, Maher D, Weil D, Espinal M, Raviglione M (2006). Targets for global tuberculosis control. Int J Tuberc Lung Dis.

[5] World Health Organization. Global tuberculosis Report. Geneva: the World Health Organization; 2011. https://apps.who.int/iris/bitstream/handle/10665/44728/9789241564380_eng.pdf?sequence=1&isAllowed=y. Accessed 14 Mar 2020.

[6] Wang L-X (2012). Analysis on the current situation of tuberculosis control in China. Chin J Public Health.

[7] Zhao F, Cheng S, He G, Huang F, Zhang H, Xu B (2013). Space-time clustering characteristics of tuberculosis in China, 2005–2011. PLoS One.

[8] The State Council Leading Group Office of Poverty Alleviation and Development. List of key counties for poverty alleviation and development. Beijing, 2012. http://www.cpad.gov.cn/art/2012/3/19/art_50_23706.html. Accessed 14 Mar 2020.

[9] National center for tuberculosis control and prevention. National TB surveillance information in 2018. Beijing; 2018.

[10] Wu B, Yu Y, Xie W, Liu Y, Zhang Y, Hu D (2017). Epidemiology of tuberculosis in Chongqing, China: a secular trend from 1992 to 2015. Sci Rep.

[11] Yu Y, Liu Y, Hu D-Y, Zhang S (2014). Epidemic characteristics of pulmonary tuberculosis in Chongqing from 2009 to 2013. Chin J Antituberc.

[12] Mao Q, Zeng C, Zheng D, Yang Y (2019). Analysis on spatial-temporal distribution characteristics of smear positive pulmonary tuberculosis in China, 2004–2015. Int J Infect Dis.

[13] Liu MY, Li QH, Zhang YJ, Ma Y, Liu Y, Feng W, et al Spatial and temporal clustering analysis of tuberculosis in the mainland of China at the prefecture level, 2005–2015. Infect Dis Poverty. 2018; 7(1): 106; 10.1016/j.ijid.2019.02.038: 10.1186/s40249-018-0490-8. 10.1186/s40249-018-0490-8 PMC6195697 30340513

[14] Tiwari N, Adhikari CM, Tewari A, Kandpal V (2006). Investigation of geo-spatial hotspots for the occurrence of tuberculosis in Almora district, India, using GIS and spatial scan statistic. Int J Health Geogr.

[15] Areias C, Briz T, Nunes C (2015). Pulmonary tuberculosis space-time clustering and spatial variation in temporal trends in Portugal, 2000-2010: an updated analysis. Epidemiol Infect.

[16] Tadesse S, Enqueselassie F, Hagos S (2018). Spatial and space-time clustering of tuberculosis in Gurage zone, Southern Ethiopia. PloS One.

[17] Zaragoza Bastida A, Hernández Tellez M, Bustamante Montes LP, Medina Torres I, Jaramillo Paniagua JN, Mendoza Martínez GD et al. Spatial and temporal distribution of tuberculosis in the state of Mexico, Mexico. Sci World J 2012; 2012: 570278; doi:10.1100/2012/570278. 10.1100/2012/570278 PMC3417174 22919337

[18] Gómez-Barroso D, Rodriguez-Valín E, Ramis R, Cano R (2013). Spatio-temporal analysis of tuberculosis in Spain, 2008-2010. Int J Tuberc Lung Dis.

[19] Wang T, Xue F, Chen Y, Ma Y, Liu Y (2012). The spatial epidemiology of tuberculosis in Linyi City, China, 2005-2010. BMC Public Health.

[20] Chongqing Bureau of Statistics. Chongqing Statistical Yearbook 2018. China statistics press. Chongqing; 2018. http://tjj.cq.gov.cn//tjnj/2018/indexch.htm. Accessed 14 Mar 2020.

[21] Chongqing Bureau of Statistics. Chongqing Statistical Bulletin on National Economic and Social Development 2018. China statistics press. Chongqing; 2018. http://www.cq.gov.cn/zqfz/gmjj/tjgb/content_398013. Accessed 14 Mar 2020.

[22] National Health commission of the People’s Pepublic of China. Notification of an adjustment to the classification of pulmonary tuberculosis reports. Beijing; 2017. http://www.nhc.gov.cn/jkj/s3589/201903/d779ae48db6446c28d1f5371ef09f5ab.shtml. Accessed 14 Mar 2020.

[23] Ge E, Zhang X, Wang X, Wei X (2016). Spatial and temporal analysis of tuberculosis in Zhejiang Province, China, 2009-2012. Infect Dis Poverty.

[24] Huang L, Li XX, Abe EM, Xu L, Ruan Y, Cao CL (2017). Spatial-temporal analysis of pulmonary tuberculosis in the northeast of the Yunnan province, People's Republic of China. Infect Dis Poverty.

[25] Huo XN, Li H, Sun DF, Zhou LD, Li BG (2012). Combining geostatistics with Moran’s I analysis for mapping soil heavy metals in Beijing, China. Int J Environ Res Public Health.

[26] Huo XN, Zhang WW, Sun DF, Li H, Zhou LD, Li BG (2011). Spatial pattern analysis of heavy metals in Beijing agricultural soils based on spatial autocorrelation statistics. Int J Environ Res Public Health.

[27] Anselin L, Syabri I, Kho Y (2005). GeoDa: an introduction to spatial data analysis. Geogr Anal.

[28] Getis A, ORD JK. The analysis of spatial association by distance statistics. Geograph Analys. 1995;27:93–115.

[29] Robertson C, Nelson TA (2010). Review of software for space-time disease surveillance. Int J Health Geogr.

[30] Kulldorff M, Athas WF, Feurer EJ, Miller BA, Key CR (1998). Evaluating cluster alarms: a space-time scan statistic and brain cancer in Los Alamos, New Mexico. Am J Public Health.

[31] Waller LA, Gotway CA. Spatial Clusters of Health Events: Point Data for Cases and Controls Applied Spatial Statistics for Public Health Data. 2004. 10.1002/0471662682.ch6.

[32] Huang L, Abe EM, Li XX, Bergquist R, Xu L, Xue JB (2018). Space-time clustering and associated risk factors of pulmonary tuberculosis in Southwest China. Infect Dis Poverty.

[33] General Office of Chongqing Municipal People’s Government. Tuberculosis Prevention and Control Plan in Chongqing from 2001 to 2010. 2002. https://law.lawtime.cn/d431406436500.html. Accessed 14 Mar 2020.

[34] Chen W, Xia Y-Y, Li T (2016). Analysis for the GiobaI and China TB epidemic situation in 2015. J Tuberc Lung Health.

[35] Thorpe LE, Frieden TR, Laserson KF, Wells C, Khatri GR (2004). Seasonality of tuberculosis in India: is it real and what does it tell us?. Lancet.

[36] Cui Z, Lin D, Chongsuvivatwong V, Zhao J, Lin M, Ou J (2019). Spatiotemporal patterns and ecological factors of tuberculosis notification: a spatial panel data analysis in Guangxi, China. PLoS One.

[37] Wubuli A, Li Y, Xue F, Yao X, Upur H, Wushouer Q (2017). Seasonality of active tuberculosis notification from 2005 to 2014 in Xinjiang, China. PloS One.

[38] Leung CC, Yew WW, Chan TY, Tam CM, Chan CY, Chan CK (2005). Seasonal pattern of tuberculosis in Hong Kong. Int J Epidemiol.

[39] Liao CM, Hsieh NH, Huang TL, Cheng YH, Lin YJ, Chio CP (2012). Assessing trends and predictors of tuberculosis in Taiwan. BMC Public Health.

[40] Kirby RS, Delmelle E, Eberth JM (2017). Advances in spatial epidemiology and geographic information systems. Ann Epidemiol.

[41] Zhou XN, Yang GJ, Yang K, Li SZ (2011). Progress and trends of spatial epidemiology in China. Chin J Epidemiol.

[42] Li XX, Wang LX, Zhang J, Liu YX, Zhang H, Jiang SW (2014). Exploration of ecological factors related to the spatial heterogeneity of tuberculosis prevalence in P. R China Global Health Action.

[43] Sun W, Gong J, Zhou J, Zhao Y, Tan J, Ibrahim AN (2015). A spatial, social and environmental study of tuberculosis in China using statistical and GIS technology. Int J Environ Res Public Health.

[44] Beiranvand R, Karimi A, Delpisheh A, Sayehmiri K, Soleimani S, Ghalavandi S (2016). Correlation assessment of climate and geographic distribution of tuberculosis using geographical information system (GIS). Iran J Public Health.

[45] Chan-yeung M, Yeh AG, Tam CM, Kam KM, Leung CC, Yew WW (2005). Socio-demographic and geographic indicators and distribution of tuberculosis in Hong Kong: a spatial analysis. Int J Tuberc Lung Dis.

[46] Shetty N, Shemko M, Vaz M, D'Souza G (2006). An epidemiological evaluation of risk factors for tuberculosis in South India: a matched case control study. Int J Tuberc Lung Dis..

[47] Munch Z, Van Lill SW, Booysen CN, Zietsman HL, Enarson DA, Beyers N (2003). Tuberculosis transmission patterns in a high-incidence area: a spatial analysis. Int J Tuberc Lung Dis.

[48] Baker M, Das D, Venugopal K, Howden-Chapman P (2008). Tuberculosis associated with household crowding in a developed country. J Epidemiol Community Health.

[49] Gustafson P, Gomes VF, Vieira CS, Rabna P, Seng R, Johansson P (2004). Tuberculosis in Bissau: incidence and risk factors in an urban community in sub-Saharan Africa. Int J Epidemiol.

[50] Cohen A, Mehta S (2007). Pollution and tuberculosis: outdoor sources. PLoS Med.

